# A Dynamic Quantitative Systems Pharmacology Model of Inflammatory Bowel Disease: Part 2 – Application to Current Therapies in Crohn’s Disease

**DOI:** 10.1111/cts.12850

**Published:** 2020-08-21

**Authors:** Katharine V. Rogers, Steven W. Martin, Indranil Bhattacharya, Ravi Shankar Prasad Singh, Satyaprakash Nayak

**Affiliations:** ^1^ Biologics Development Sciences Janssen Biotherapeutics Janssen Research and Development, LLC Spring House Pennsylvania USA; ^2^ Pharmacometrics Global Clinical Pharmacology Pfizer Inc. Cambridge Massachusetts USA; ^3^ Clinical Pharmacology and Pharmacometrics Takeda Pharmaceuticals Cambridge Massachusetts USA; ^4^ Clinical Pharmacology Early Clinical Development Pfizer Inc. Cambridge Massachusetts USA

## Abstract

Inflammatory bowel disease (IBD) is a heterogeneic disease with a variety of treatments targeting different mechanisms. A multistate, mechanistic, mathematical model of IBD was developed in part 1 of this two‐part article series. In this paper, application of the model to predict response of key clinical biomarkers following different treatment options for Crohn’s disease was explored. Five therapies, representing four different mechanisms of action, were simulated in the model and longitudinal profiles of key clinical markers, C‐reactive protein and fecal calprotectin were compared with clinical observations. Model simulations provided an accurate match with both central tendency and variability observed in biomarker profiles. We also applied the model to predict biomarker and clinical response in an experimental, combination therapy of existing therapeutic options and provide possible mechanistic basis for the increased response. Overall, we present a validated, modular, mechanistic model construct, which can be applied to explore key biomarkers and clinical outcomes in IBD.

We developed a quantitative systems pharmacology (QSP) model for inflammatory bowel disease (IBD), which is discussed in part 1, “A Dynamic Quantitative Systems Pharmacology Model of Inflammatory Bowel Disease: Part 1 – Model Framework.” In this second part (part 2), we present a comprehensive evaluation of model performance using clinically observed biomarker changes, following administration of different treatments, either approved for IBD or under clinical development.

Novel molecules targeting a varied number of mechanisms are being developed in the IBD space. However, no compound to date has been shown to be efficacious in a high proportion of patients and, therefore, an unmet need for new drugs remains in this area, especially considering the wide variability in clinical response and loss of efficacy observed over time for many treatments. The large number of target mechanisms being tested in IBD as well as the low response rate to specific drugs calls for novel methods to predict drug efficacy, and identifying the right patient subsets would have significant drug development and clinical impact. Nonresponse is common even with approved drugs, such as anti‐TNFα, in which ~ 10–30% of patients are initial treatment nonresponders and an additional 23–46% of patients lose response over time, and this can be attributed to immunogenicity response.^1^ In addition, many cytokine inhibitors have been tested and failed in clinical trials for IBD, including anti‐IL17 (secukinumab and brodalumab), anti‐IFNγ (fontolizumab), and anti‐IL13 (anrukinzumab and tralokinumab).^2^ Our QSP model, because of its mechanistic basis and dynamic nature, is an ideal computational platform that could be utilized to test new drug targets, determine possible mechanisms for treatment failures, test hypothesis of drug schedules or combinations, and predict treatment response in distinct subpopulations of patients with IBD.

A mechanistic QSP model was developed (part 1) from literature and in‐house data, which captured the response of key biomarkers at steady‐state in Crohn’s disease (CD) and ulcerative colitis using a common model structure. In addition, we performed parameter sensitivity analysis to identify key mechanisms affecting fecal calprotectin (FCP) and C‐reactive protein (CRP) and applied the model to understand mechanisms behind worsening of CD in case of IL‐17 inhibition. In this paper, we assess the impact of underlying biological structure through modulation of multiple mechanisms by treatments either approved or in development for CD, such as TNFα, IL‐12p40, IL‐23p19, and IL‐6, and novel drug combinations. We specifically utilize our CD model, developed in part 1, to simulate multiple therapies and compare published biomarker outcomes, create and test hypotheses with treatment biomarker responders, and predict clinical biomarker response for combination therapies *in silico*.

## METHODS

The model structure and the definition of the CD model are defined in the part 1 paper. The model was built using MatLab’s Simbiology software.

### Simulation of virtual populations against various treatments: Model validation

Treatments included anti‐TNFα (infliximab), anti‐IL‐12p40 (ustekinumab), anti‐IL‐23 (risankizumab and brazikumab), and anti‐IL‐6 (PF‐04236921). For each treatment, first a baseline population from our total 40,000 plausible patients was selected to match the CRP and FCP distribution of the clinical trial that was being simulated (**Figure** **S9** shows an example for ustekinumab). This was necessary as baseline values between clinical trials were highly variable and the published results did not have patient level data to compare. In all cases, the virtual population response was compared against induction clinical trials in the CD population. The drug concentrations were modeled as two‐compartment pharmacokinetic (PK) models using published PK parameters. The PK parameters were assumed to be equivalent in all patients for this study. For each case model simulations were compared with the corresponding metric from published clinical data (i.e., median response, mean response, median absolute values, etc.).

The ustekinumab trial used was the UNITI‐2 clinical trial in CD, where anti‐TNFα naïve patients were given a single i.v. dose of placebo, 130 mg, or 6 mg/kg dose (assumed as 420 mg in the model; *n* = 210, *n* = 209, and *n* = 209).^3^ The brazikumab trial used was a phase IIa study in patients with moderate to severe CD (Crohn’s Disease Activity Index (CDAI) 220–450), in which patients in the treatment group received 700 mg brazikumab i.v. at weeks 0 and 4 (*n* = 59).^4^ The following open label period was s.c. 210 mg doses every 4 weeks post week 12. For the risankizumab clinical data, published results were used from a phase II trial in moderate to severe CD (CDAI range 220–450), in which patients received placebo, 200 mg, or 600 mg doses i.v. at 0, 4, and 8 weeks (*n* = 39, *n* = 41, and *n* = 41).^5^ We looked at an infliximab study to obtain clinical data for CRP and cytokines in patients with CD. The subjects in this study received 5 mg/kg i.v. dose at weeks 0, 2, and 6 (*n* = 22).^6^ The FCP values were from a study in which infliximab was dosed at 5 mg/kg i.v. at weeks 0 and 8 (*n* = 14).^7^ The PF‐04236921 (ANDANTE I) was a phase II induction trial in patients with moderate to severe CD, in which placebo, 10, 50, or 200 mg s.c. was given on days 1 and 28 (*n* = 69, *n* = 67, *n* = 71, and *n* = 40).^8^

## RESULTS

Specific applications of the model from part 1 were evaluated for CD and the results are discussed below. Assessments included longitudinal CD biomarker response (CRP and FCP), population outcomes, and clinical biomarker response for combination treatments. The model in part 2 additionally considered drug PKs and drug binding to the target, while keeping all the other parameters from part 1 unchanged.

### Incorporating different treatment mechanisms in the model

**Figure 1:**
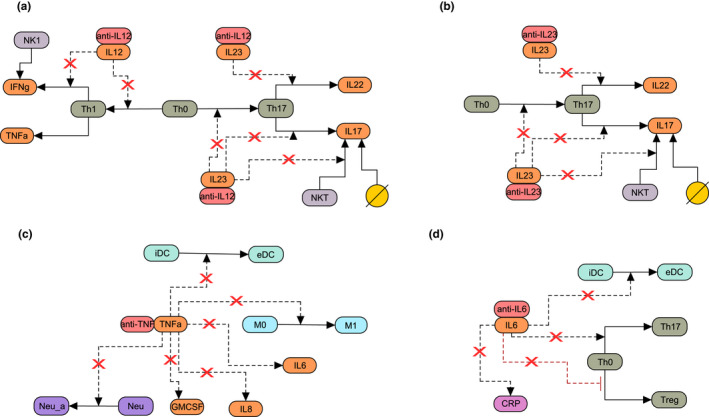
Schematic of drug mechanisms added to the Crohn’s disease model. Shown is the effect of (**a**) anti‐IL‐12p40 (ustekinumab), (**b**) anti‐IL‐23 (brazikumab and risankizumab), (**c**) anti‐TNFα (infliximab), and (**d**) anti‐IL‐6 on biological interactions included in the model. Dotted lines indicate that the species in the model influences the reaction, solid lines indicate either differentiation or production, the yellow circle denotes alternative sources of IL‐17, and red x denote downregulation of the interaction due to treatment. eDC, effector dendritic cells; iDC, immature dendritic cells;M0, resting macrophages; M1, classically activated macrophages; Neu, neutrophils; Neu_a, activated neutrophils; NK 1, natural killer 1; Th0, T naïve; Th1, T helper 1; Th17, T helper 17; Treg, T regulatory.

The following different treatments, anti‐IL‐12p40 (ustekinumab), anti‐IL‐23 (risankizumab, brazikumab), anti‐TNFα (infliximab), anti‐IL‐6 (PF‐04236921) were incorporated in the model via a binding reaction to the specific cytokine, shown in **Figure** **1**. These drugs were chosen as they target different mechanisms of action in CD and the choice of treatments for simulation was also based on the availability of clinical biomarker data in response to treatment. To accurately model the longitudinal time course of treatment effect, the plasma PK of the drug was simulated based on published information and used as an input to the systems model (see next section for details). To estimate the variability in treatment response, a virtual population of CD subjects was also generated for each simulated drug and response to the drug was modeled at an individual level (details regarding virtual population generation is provided in the next section).

**Figure** **1** shows the effect of different mechanisms of action of approved or in‐development therapies in CD. Ustekinumab is an IgG2 monoclonal antibody against IL‐12p40 and blocks the activity of the interleukins IL‐12 and IL‐23 leading to decreased differentiation of naïve T cells to Th17 and Th1. This decreased differentiation of T cells, in turn, causes a decrease in IL‐17 and IFNγ levels (**Figure** **1** **a**). Anti‐IL‐23 antibodies simulated in the model (brazikumab and risankizumab), on the other hand, bind to the p19 subunit of IL‐23, and block the differentiation of naïve T cells to Th17 and decrease IL‐17 levels (**Figure** **1** **b**). Infliximab is an anti‐TNFα antibody, which binds to TNFα and prevents activation of dendritic cells, macrophages, and neutrophils, and leads to decreased levels of IL‐6, IL‐8, and GM‐CSF (**Figure** **1** **c**). Finally, PF‐04236921 binds to IL‐6 and decreases the differentiation of naïve T cells to Th17, as well as reducing the activation of dendritic cells and the production of IL‐6 (**Figure** **1** **d**).

### Generating selective virtual patient populations based on baseline biomarker levels

When comparing placebo corrected response across various randomized clinical trials with different types of treatments for CD, treatment effect tends to show a wide range of responses.^9^ There may be multiple reasons for these differences in response rates and biomarker response in CD trials, including trial design, patient population under study, or both.^10^ To delineate the contribution of patient population on the varied response, one approach is to simulate the impact of baseline patient characteristics. For this, a virtual patient population was selected to mimic the baseline characteristic for each treatment case and was achieved using the following steps.

**Figure 2:**
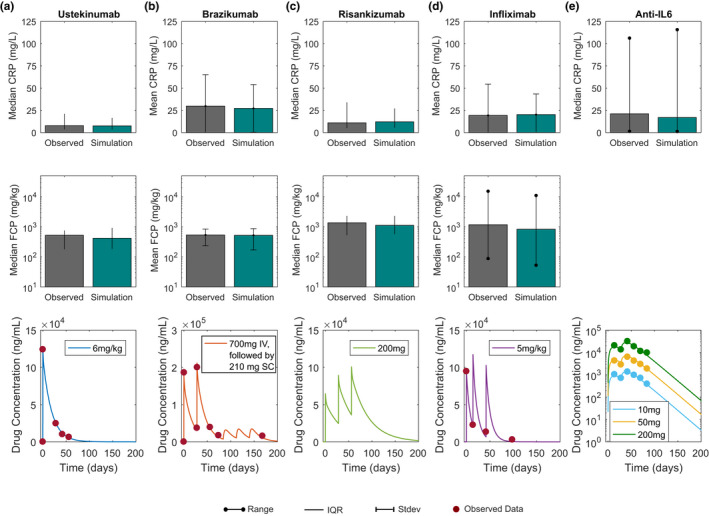
Simulated and observed CRP and FCP baseline levels and drug concentration time profiles following single or multiple dose administration of various CD treatments in development or approved. Column (**a**) shows the median baseline concentrations of CRP and FCP and ustekinumab concentration time profiles following single i.v. dose of 6 mg/kg. Column (**b**) shows the mean CRP and FCP baseline and drug concentration time profile for 700 mg brazikumab i.v. given at time zero and day 28, followed by once every 8 week dose of 210 mg SC. Column (**c**) shows median baseline CRP and FCP and simulated drug concentration time profile of risankizumab following once every 28 day administration of 200 mg i.v. Column (**d**) shows mean CRP, median FCP baseline levels, and infliximab concentration time profile for 5 mg/kg IV at 0, 2, and 6 weeks. Column (**e**) shows median baseline CRP (50 mg population) and drug concentration time profiles for three doses (10, 50, and 200 mg) PF‐04236921 given s.c. at 0 and 4 weeks. Circles indicate published data for each trial. Grey bars indicate published results of median or mean and green indicates virtual population results. Lines indicate interquartile range (IQR), bars SD, and lines with dots are range. FCP for IL‐6 is excluded due to high variability in data.

**Figure 3:**
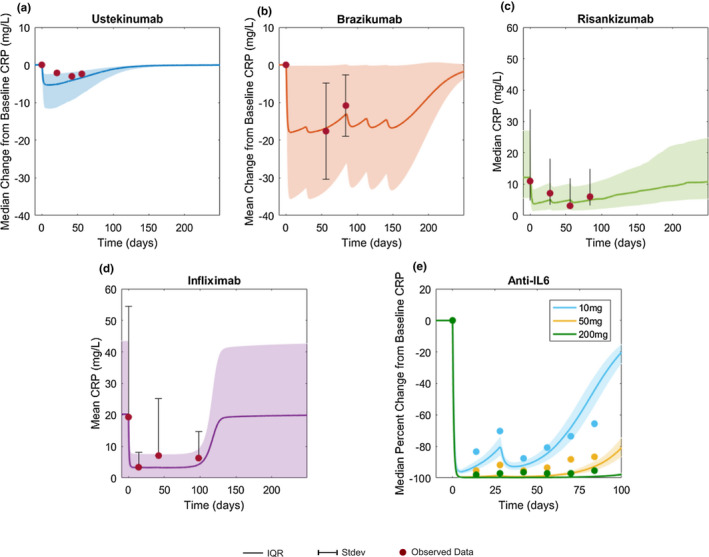
Simulation versus observed CRP concentration profiles. CRP change from baseline after treatment with (**a**) ustekinumab and (**b**) brazikumab. Absolute CRP concentration time profiles following (**c**) risankizumab and (**d**) infliximab treatment, respectively. (**e**) Median percent change from baseline following anti‐IL‐6 treatment, PF‐04236921. Solid lines indicate mean or median and shaded area indicates interquartile range (IQR; observed data = lines) or SD (observed data = bars) of the virtual population. Circles indicate published data for each trial. The UNITI‐2 trial did not publish CRP variability and IQR was assumed for model simulation. For all treatment dosing information used, refer to **Figure** **2**.

**Figure 4:**
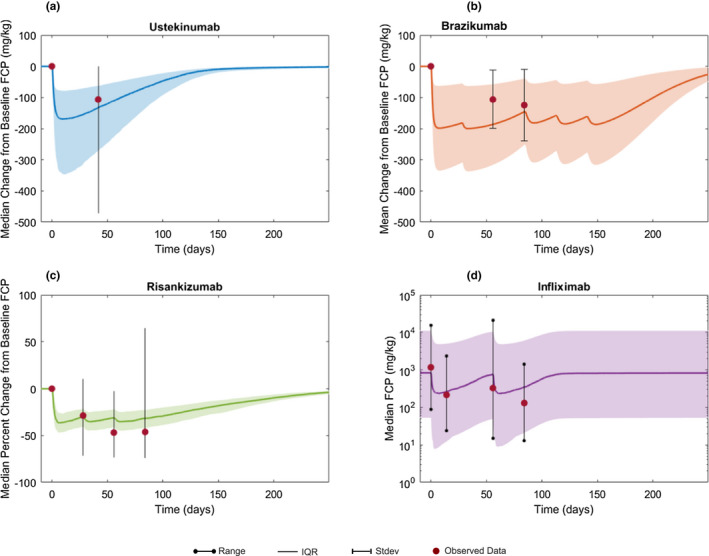
Simulation vs. observed FCP concentration time profiles. Change from baseline in FCP concentration following (**a**) ustekinumab and (**b**) brazikumab administration, respectively. (**c**) Median percent change from baseline FCP after risankizumab treatment. (**d**) Median absolute value of FCP following infliximab treatment. Solid lines indicate mean or median and shaded area indicates interquartile range (IQR; observed data = lines), SD (observed data = bars), or range (observed data = lines with dots). Circles indicate published data for each trial. For all treatment dosing information used, refer to **Figure** **2**.

**Figure 5:**
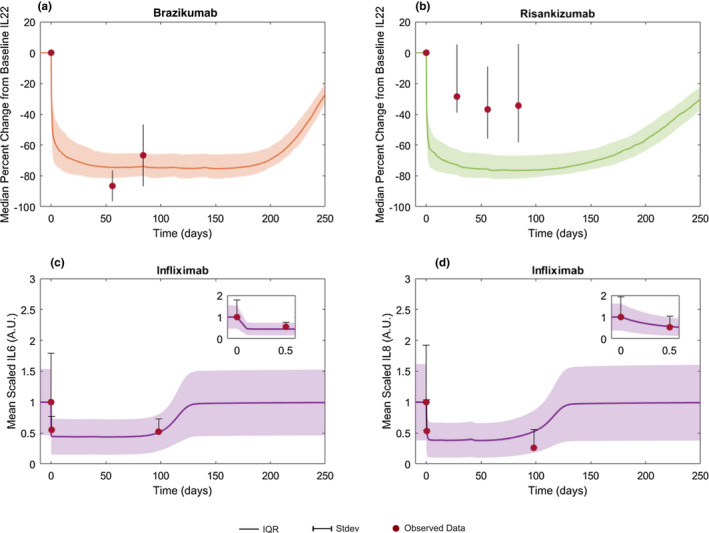
Simulation vs. observed biomarker profiles. Median percent change from baseline serum IL‐22 following treatment with two anti‐IL‐23 inhibitors, (**a**) brazikumab and (**b**) risankizumab. Mean scaled serum (**c**) IL‐6 and (**d**) IL‐8 following anti‐TNFα, infliximab. Solid lines indicate mean or median and shaded area indicates interquartile range (IQR; observed data = lines) or SD (observed data = bars). Scaled values are normalized by the baseline mean population value. Circles indicate published data for each trial. For all treatment dosing information used, refer to **Figure** **2**.

Step 1: Virtual patients were selected from the database of 40,000 plausible patients with CD (see part 1) to resemble both baseline CRP and FCP statistics (see the Methods section) reported in each clinical trial (**Table** **S1**). **Figure** **2** shows bar charts of baseline CRP (top row) and FCP (middle row) levels reported for each of the clinical studies along with the model predicted values for the virtual population selected for the study. Here, each column (from A to E) shows the steady‐state CRP, FCP, and simulated PK for each mechanism of action simulated in the model. The reported statistic (mean and median) and variance (interquartile range, SD, and range) differs between clinical trials and, therefore, each comparison was made against the reported statistic (**Figures** **2**, **3**, **4**, **5**).

Step 2: To model the time course of various drug treatment effects, PK models were developed based upon published PK parameters (clearance and volume of distribution). Further, to assess the adequacy of the PK models, simulations of the time course of serum concentrations of drug were performed for each compound and compared with reported concentrations, and are shown in **Figure** **2** (bottom row). Because we were interested in understanding the pharmacodynamic variability occurring due to differences in underlying biological parameters, all subjects were assumed to have an identical serum concentration time profile, thus the variability seen in biomarker response is solely due to differences in system parameter values between virtual subjects. As seen from the panels in **Figure** **2** where each column describes the CRP, FCP, and PK behavior for one treatment mechanism, a single model can describe the steady‐state biomarker behavior in therapies, which are markedly different from each other in their mechanisms of action.

### Model prediction of CRP, FCP, and biomarker response to treatments

The observed and model predicted longitudinal change in CRP following different treatments is shown in **Figure** **3**. The model adequately predicted a minor change in CRP concentrations from baseline following treatment with ustekinumab (**Figure** **3** **a**). For brazikumab, the model not only predicted the placebo corrected least square mean change in CRP but captured the uncertainty as well (**Figure** **3** **b**). For risankizumab, the model simulation predicted a drop in median absolute CRP levels after treatment in the 200 mg group, which is supported by the data from the clinical study (**Figure** **3** **c**). The model predicted a large decrease in serum CRP levels following infliximab administration (**Figure** **3** **d**), similar to the trend seen in observed CRP values. The model also captured the dose‐response relationships for CRP suppression observed following IL‐6 inhibitor PF‐04236921 (**Figure** **3** **e**). The median percentage change in CRP, which is a pharmacodynamic marker of IL‐6 suppression, was 95% in the 200 mg dose, 86.3% in the 50 mg dose, and 65.6% in the 10 mg dose at week 12 from the study.^8^ The corresponding model predicted values for CRP suppression from the model are 99% (200 mg dose), 92.5% (50 mg dose), and 46% (10 mg dose). IL‐6 inhibition led to the largest decrease in CRP in the model as CRP production is directly modulated by IL‐6 in the model, whereas anti‐IL‐12p40 with the ustekinumab UNITI2 population led to the smallest decrease in CRP. This may be due to a baseline population effect when comparing the ustekinumab and brazikumab trials because the UNITI2 trial had the lowest starting level of CRP (**Figure** **S7** predicts only a small difference in maximum CRP reduction between the 2 drugs when the same population is considered).

**Figure** **4** shows that the model predicted changes in FCP following various treatments, ustekinumab (**Figure** **4** **a**), brazikumab (**Figure** **4** **b**), risankizumab (**Figure** **4** **c**), and infliximab (**Figure** **4** **d**). Fecal calprotectin change following anti‐IL‐6 was excluded due to the high variability reported in observed data.^8^ The median change in FCP concentration (**Figure** **4** **a**) following a single 6 mg/kg dose of ustekinumab is accurately predicted by the model. For brazikumab, the model seems to slightly overestimate the placebo corrected least square mean change in FCP, although there is considerable variability in FCP change, which overlaps with model predicted results (**Figure** **4** **b**). In case of risankizumab, the median percent change in FCP levels is similar to those reported in the 200 mg dose group, with less variability in the model prediction (**Figure** **4** **c**). The simulated median and range FCP response after two doses of infliximab is shown (**Figure** **4** **d**).

Although CRP and FCP are commonly reported in clinical studies of CD, the availability of published data for additional biomarkers was limited and often limited to key cytokines related to the mechanism of action for the various treatments. Time course profiles of cytokines IL‐22, IL‐6, and IL‐8 were simulated and compared with literature data when available. **Figure** **5** shows a large decrease from baseline in model predicted IL‐22 concentrations following treatment with the IL‐23 inhibitors brazikumab (**Figure** **5** **a**) and risankizumab (**Figure** **5** **b**). A smaller change in IL‐22 was observed following risankizumab vs. brazikumab administration, however, the model was not able to distinguish the difference in this IL‐22 effect size between the two molecules (**Figure** **5** **b**). The model accurately predicted the rapid decrease in both IL‐6 and IL‐8 following infliximab administration (**Figure** **5** **b,c**). We also simulated the additional doses reported in these studies and the results for those doses can be found in **Figures** **S1–S3**. Thus, the combined results from **Figures** **3**, **4**, **5** show that the model can accurately match average longitudinal profiles of key cytokines and biomarkers of inflammation. In addition, the observed variability for a variety of different treatments, when initialized with the right study population, was also accurately captured by the model.

### Prediction of responder population

There is a need to identify patient characteristics that influence clinical response in patients with IBD. Different approaches have been proposed, including biomarker stratification at trial enrollment or biomarker use in dose adjustment while the study is ongoing.^11^ A QSP model could provide an advantage in this regard as it is uniquely suited to help in gaining insight into whether different subgroups of patients can be identified based on the clinical response. We used the current model to identify and understand differences in response based on the important clinical biomarkers, CRP and FCP. However, an important point to be noted is that although CRP and FCP are considered important in CD, the quantitative relationship between the markers and the clinical score (e.g., Crohn’s Disease Activity Index (CDAI), is not well established. To compare the clinical efficacy response to drug treatments, we used cutoffs based on biomarker values as a surrogate for clinical response. These cutoff values were based on literature information. Three case scenarios defining response based on cutoffs defined by percentage reduction in CRP, absolute CRP value, and an absolute FCP value are discussed below.

**Figure 6:**
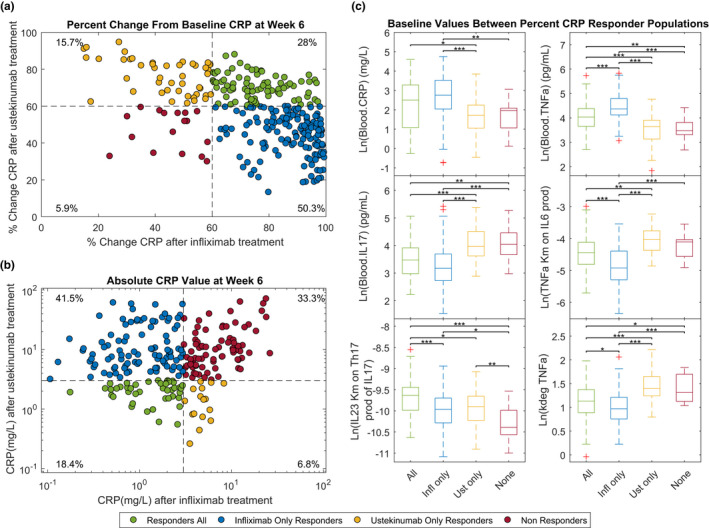
Subject level CRP response to infliximab (5 mg/kg i.v. at weeks 0, 2, and 6) and ustekinumab (single does 6 mg/kg i.v.) at week 6 in a patient with Crohn’s disease (CD). (**a**) Shows the percent change from baseline CRP and (**b**) shows the absolute CRP levels at week 6 following either infliximab or ustekinumab administration to each individual virtual patient (*n* = 286). In **b** only patients whose baseline CRP level was above 3 mg/L (*n* = 234) were included. Each dot represents a virtual patient and are colored depending upon if they were a CRP responder to both drugs (green), responders to infliximab (blue), responders to ustekinumab (orange), and nonresponders (red). Response is defined as > 60% change from baseline in the percent change case and < 3 mg/L in the absolute value case. (**c**) Shows bar charts of baseline values for various biomarkers according to the subject’s response to infliximab or ustekinumab treatment based on achieving greater than 60% change from baseline levels of CRP at week 6. IQR, interquartile range.

In the first case, the biomarker response was defined as ≥ 60% CRP decrease at week 6 in the presence of treatment.^12^ The predicted percent change in CRP response after anti‐TNFα (infliximab) or anti‐IL‐12p40 (ustekinumab) induction schedules are shown for each virtual patient (**Figure** **6** **a**). Based on the prespecified CRP cutoff, the model predicts that for the 284 virtual patients, 28% will respond to both treatments, 50.3% will respond to infliximab only, 15.7% will respond to ustekinumab only, and 5.9% will not respond to either treatment. **Figure** **6** **c** shows key baseline species and parameter values that were found to be different between responder groups (e.g., responders to infliximab only had higher levels of serum TNFα and CRP (blue, **Figure** **6** **c**), whereas those responding to only ustekinumab had higher serum levels of IL‐17 (yellow, **Figure** **6** **c**)). With regard to parameters in the model, the degradation of TNFα and the half maximum rate (Km) of TNFα on IL‐6 production is lower only in the infliximab group (blue).

In the second case, an absolute cutoff for normalized CRP of 3 mg/L at week 6 was used.^13^ In this case, virtual patients under the cutoff at baseline were excluded (50 of 284 virtual patients). The new level of CRP concentration predicted after infliximab or ustekinumab treatment for each virtual patient is shown in **Figure** **6** **b**. For an absolute CRP cutoff, the model predicts for the 234 virtual patients with high levels of CRP (3 mg/L) that 18.4% will respond to both treatments, 41.5% will respond to infliximab only, 6.8% will respond to ustekinumab only, and 33.3% will not have normalized CRP with either treatment. Understandably, when response is defined by normalized CRP concentration, the responders to either of the treatments had lower baseline levels of CRP to nonresponders (**Figure** **S4**). Alternatively, if the response cutoff is set at normalized CRP of 5 mg/L, the percentage of responders was slightly higher for the 205 virtual patients above the cutoff (24.9% will respond to both treatments, 49.3% will respond to only infliximab, 6.3% will respond to only ustekinumab, and 19.5% will not have normalized CRP in case of either treatment; **Figure** **S5**).

Finally, a third case in which an absolute cutoff for normalized FCP of 250 mg/kg as a set point for response was also analyzed.^14^ Similar to the response based on absolute CRP values, patients with baseline FCP values below the cutoff were excluded (36 of 284). The model predicts that at 6 weeks 13.3% will respond to both treatments, 15.7% will respond to infliximab, 0.4% to ustekinumab, and 70.6% will not have normalized FCP levels in either treatment case (**Figure** **S6a**). In general, FCP nonresponders had higher baseline levels of neutrophils, IL‐8, FCP, and CRP (**Figure** **S6c**).

### Combination of anti‐TNFα and anti‐IL‐12p40 treatment in CD subjects

As the number of approved therapies with very different mechanisms of action increases, there is an interest in exploring combination therapy to provide therapeutic benefit to a greater proportion of patients. Currently, there is very limited published information on the efficacy and safety data with combination treatments in IBD (e.g., Hiren *et al*. reviewed data from one randomized control trial and three case reports or series and advocated the need for more trials before general application in IBD.^15^ A phase IV trial combining vedolizumab (anti‐integrin), adalimumab (anti‐TNFα), and methotrexate in CD is currently ongoing (Identifier: NCT02764762). Another recent case series by Buer *et al*. evaluated safety and clinical response of combination anti‐TNFα and vedolizumab therapy in 10 patients with IBD and concluded that the combination treatment is safe and might represent a treatment option in selected subjects.^16^

**Figure 7:**
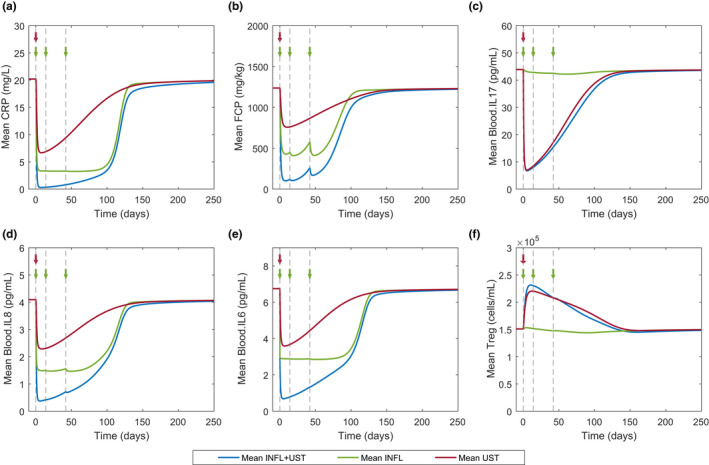
Model prediction of infliximab (INFL) and ustekinumab (UST) alone or in combination on biomarkers in patients with Crohn’s disease. Mean CRP, FCP, IL‐17, IL‐8, IL‐6, and Treg response to combination (blue), infliximab alone (green), or ustekinumab only (red) treatment. Virtual population size of 286 was used for all three treatment cases. Arrows and dotted lines denote i.v. dosing of 5 mg/kg infliximab (green) and i.v. dosing of 6 mg/kg ustekinumab (red).

Using the CD model and the population from the infliximab case, a combination therapy with the ustekinumab induction (single dose i.v. 6 mg/kg at week 0) and infliximab induction (5 mg/kg i.v. at weeks 0, 2, and 6) was tested. **Figure** **7** shows model prediction of median biomarker response after the three treatment options, each therapy alone or in combination, using the same infliximab virtual population. In all cases, the combination treatment seems to have the largest effect (i.e., decrease in clinical markers). Both median CRP and FCP decrease below the cutoffs defined above, 3 mg/L and 250 mg/kg, respectively. Thus, our model predicts that there could be potentially increased efficacy with the combination treatment of an anti‐TNFα and anti‐IL‐12p40 treatment in CD subjects who respond inadequately to ustekinumab or infliximab monotherapy. This predicted increase in effect could be due to a concomitant downregulation of both IL‐23 regulated proteins, such as IL‐17, as well as downregulation of TNFα proteins, such as IL‐8, which does not occur following anti‐IL‐12p40 or anti‐TNFα monotherapy.

## DISCUSSION

QSP modeling is increasingly being applied to understand complex diseases as it can provide a comprehensive quantitative view of disease biology in a single model construct. Recently, a QSP model of bone formation in osteoporosis was also applied in a regulatory setting to further optimize dose regimen, pointing toward the increased application of QSP modeling in clinical drug development.^17^ IBD poses a unique challenge for QSP modeling as it is highly dependent on available biomarker information and such information is both scarce and is quite variable.^18^ In this two‐part series of papers, we present a model of IBD, which includes the main cytokines/interleukins and cell‐types implicated in IBD, trained it with steady‐state levels of key biomarkers, and provide a comparison of clinical markers with existing therapies. For example, the QSP model for CD was able to match the response of biomarkers, such as CRP and FCP to anti‐IL‐12p40, anti‐IL‐23, anti‐TNFα, and anti‐IL‐6 therapy. For these predictions, the model was modified to account for the additional biology needed to describe drug binding to its target as well as the PKs of the drug and matching of baseline CRP and FCP data, while all the remaining parameters in the original model were unchanged (see part 1).

The current model has been developed in a modular fashion to allow for easy inclusion/exclusion of biological mechanisms, hypothesis generation in terms of patient nonresponders defined by biomarkers, as well as to test hypothesis on combination treatments. The model prediction of 25.2% anti‐TNFα naïve patients achieving CRP normalization of 3 mg/L after ustekinumab treatment is similar to the UNITI‐2 trial in which 30.9% of patients saw CRP normalization^3^ (placebo response = 5.6%). The model underpredicts the number of patients that would have normalized FCP levels ≤ 250 mg/kg with ustekinumab treatment compared with the UNITI‐2 trial (i.e., the model predicted response rate is 13.7% as compared with the observed response rate of 30.4%). However, it is to be noted that the placebo response in this study was 15.7%, so the placebo corrected response rate of 14.7% is closer to the model predicted value. A key point to note is that although the QSP model predicts treatment effect, it does not take into account the impact of study design on treatment effect, which has been suggested to impact the placebo response in IBD trials.^11^ We then applied the model to test whether a combination of infliximab and ustekinumab would lead to a greater response rate in terms of biomarkers. In all the simulated cases, combination treatment led to a greater decrease in CRP concentration (see individual level CRP response in **Figure** **S8**). There is existing preclinical evidence to support this hypothesis (e.g., a bispecific TNFα and IL‐17 antibody in a fibroblast cell line showed that the levels of IL‐6, IL‐8, and granulocyte colony‐stimulating factor have a significant decrease compared with monotherapy.^19^ To our knowledge, this is the first comprehensive, mechanistic, ordinary differential equation‐based model of IBD, which can match a diverse variety of biomarker response and can predict responder rates based on biomarker cutoffs matching with clinical observations. However, there are still a few gaps in the current model that would need to be addressed in future versions.

This current version of the QSP model is limited to mechanisms that were considered important in the literature and were biologically relevant to treatments to keep the model complexity manageable without sacrificing key biological mechanisms. Future model improvements could include adding mechanistic biology to better describe the effect of drugs (e.g., the target proteins for ustekinumab (anti‐IL‐12p40) and the anti‐IL‐23 inhibitors, IL‐12 and IL‐23, respectively, are represented as single proteins in the model, but are actually heterodimeric cytokines); IL‐12 is a dimer of IL‐12p35 and IL‐12p40 and IL‐23 is a dimer of IL‐12p40 and IL‐23p19.^20^ Another simplification in the model is related to TNFα biology, as TNFα exists in both soluble and membrane‐bound forms, and there are two receptors TNF‐R1 and TNF‐R2 with two distinct intracellular pathways.^21^ This biology may be important in comparing modalities of TNFα inhibitors as the drugs targeting soluble TNF receptors (instead of anti‐TNF antibodies; i.e., etanercept and onercept, saw no efficacy in clinical trials of CD).^21^ Similar to the disease‐related biology, simplifications were also made to the treatment mechanism of action (e.g., additional mechanisms of anti‐TNFαs). For infliximab, mechanisms not considered in the simulated treatment include induction of lamina propria T cell apoptosis as well as induction of M2 cells.^21^ With regard to modeling IL‐6 biology, IL‐6 signaling is more complicated than the mechanisms included in the model. A more detailed mechanism describing the two types of IL‐6 signaling (trans and classical), soluble IL‐6R binding to IL‐6, and intracellular signaling involving Jak/STAT3 can be considered and has been modeled in detail by Dwivedi *et al*.^22^ Incorporation of IL‐6 signaling details may be able to explain the slight overprediction of CRP response seen in the model predictions for anti‐IL‐6 treatment.

A major component of IBD not included in the model, is related to epithelial biology, extensive Th2 downstream biology, and cell transport. Understanding intestinal epithelial damage and healing would be important in cases where the treatment is thought to effect epithelial integrity (i.e., anti‐IL‐17 inhibition) and in linking endoscopic score to epithelial healing. Barrier health is also considered to be a factor in modulating FCP levels as neutrophils and calprotectin entering the lumen through a damaged barrier impact FCP levels, apart from neutrophils present in the gut.^23^ Barrier health and gut bacteria may also have an effect on treatment efficacy as anti‐inflammatory therapies are potent immunosuppressants whose application can lead to exacerbation of lesions/infection.^24^ Another important aspect of the disease not considered in the current model, is related to its location, which also plays a large role in clinical response and outcome,^14^ FCP levels may also be related to disease location, which is not considered (i.e., colonic, ileal, or ileocolic) in the current version.^25^

An eventual outcome of the model would be to translate biomarker response to clinical score. However, there are plenty of challenges currently with reliably associating biomarker changes with clinical disease scores, such as CDAI, as they include subcomponents that are not directly related to biomarker changes (e.g., abdominal pain and general well‐being). In addition, there are multiple clinical scores in CD trials, Harvey Bradshaw, CD Endoscopic Index of Severity (CDEIS), Simple Endoscopic Score for CD (SES‐CD), and Patient Reported Outcomes, such as IBDQ, making studies difficult to compare.^14^ The model can predict biomarker response and involves extensive aggregation of information from multiple clinical trials, preclinical studies, and longitudinal natural history studies; however, other information, such as individual drug concentrations or other laboratory tests, may be necessary to predict clinical response. With vigorous validation and model improvement and with more biological data becoming available, we hope that our mechanistic model of IBD can potentially be used along with other quantitative approaches to gain a better understanding biomarker response and its relationship to clinical response to design more effective studies in the IBD space.

## Funding

This work was sponsored by Pfizer.

## Disclosure

This work was sponsored by Pfizer.

## Conflict of Interest

At the time this work was done, K.V.R. and I.B. were employees of Pfizer. S.W.M., R.S.P., and S.N. are current employees of Pfizer Inc. This work was sponsored by Pfizer.

## Author Contributions

K.V.R. and S.N. wrote the manuscript. K.V.R., S.M., I.B., R.S.P.S., and S.N. designed the research. K.V.R. performed the research. K.V.R. analyzed the data.

**Study Highlights**

**WHAT IS THE CURRENT KNOWLEDGE ON THE TOPIC?**

For a complex disease, such as inflammatory bowel disease (IBD), it is quite challenging to reliably predict the behavior of key biomarkers in response to an interventional therapy.

**WHAT QUESTION DID THIS STUDY ADDRESS?**

This study provides a basis to understand the behavior and variability we observe for new therapies and combination treatments in IBD. It also provides clues for mechanistic reasons behind failure of previous treatment modalities.

**WHAT DOES THIS STUDY ADD TO OUR KNOWLEDGE?**

This model can simulate the effects of wide variety of existing as well as future potential treatments (e.g., combination therapies) and match the behaviors of key clinical biomarkers. It can also provide predictions regarding clinical efficacy based on biomarker behavior in the IBD population.

**HOW MIGHT THIS CHANGE CLINICAL PHARMACOLOGY OR TRANSLATIONAL SCIENCE?**

The model provided in this study is modular and can be extended to include specific biological mechanisms of interest. It provides a way to generate accurate predictions regarding clinical efficacy and clinical markers for future therapies in the IBD population.

## Supporting information

Figure S1‐S10

Table S1

SupplementaryMaterial S1

SupplementaryMaterial S2
