# Supplementary material for: A Dynamic Quantitative Systems Pharmacology Model of Inflammatory Bowel Disease: Part 2 – Application to Current Therapies in Crohn’s Disease
Source: Clin Transl Sci. 2020 Aug 21;14(1):249–59. doi: 10.1111/cts.12850 (PMC7877864; doi:10.1111/cts.12850)
Supplement: Supplementary file 1 — Figure S1‐S10 [file CTS-14-249-s001.pdf]

Supplementary Figure 1 (S1)

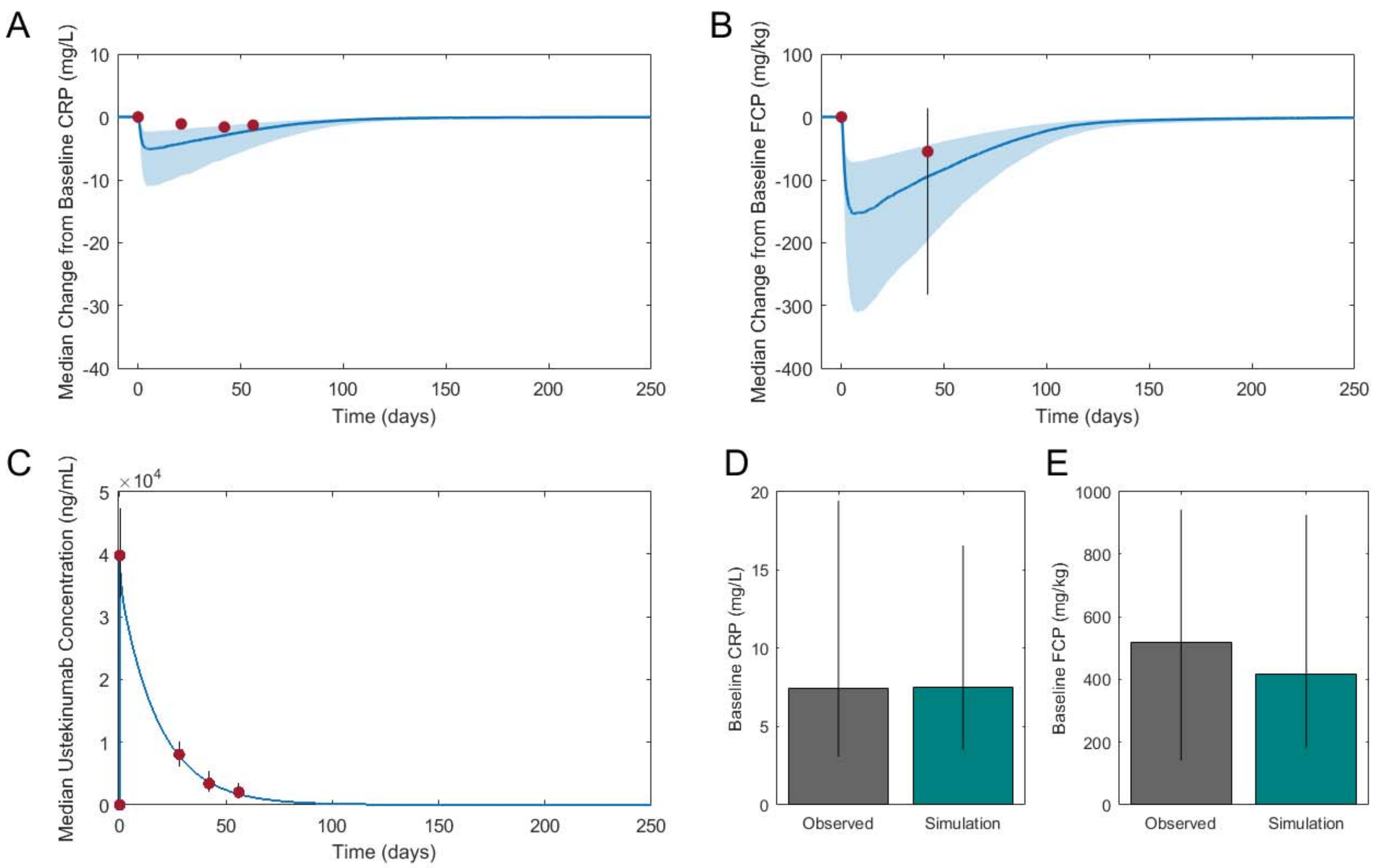

Figure S1: Concentration time profiles (CRP, FCP, ustekinumab) in response to a dose of ustekinumab (130 mg).

Supplementary Figure 2 (S2)

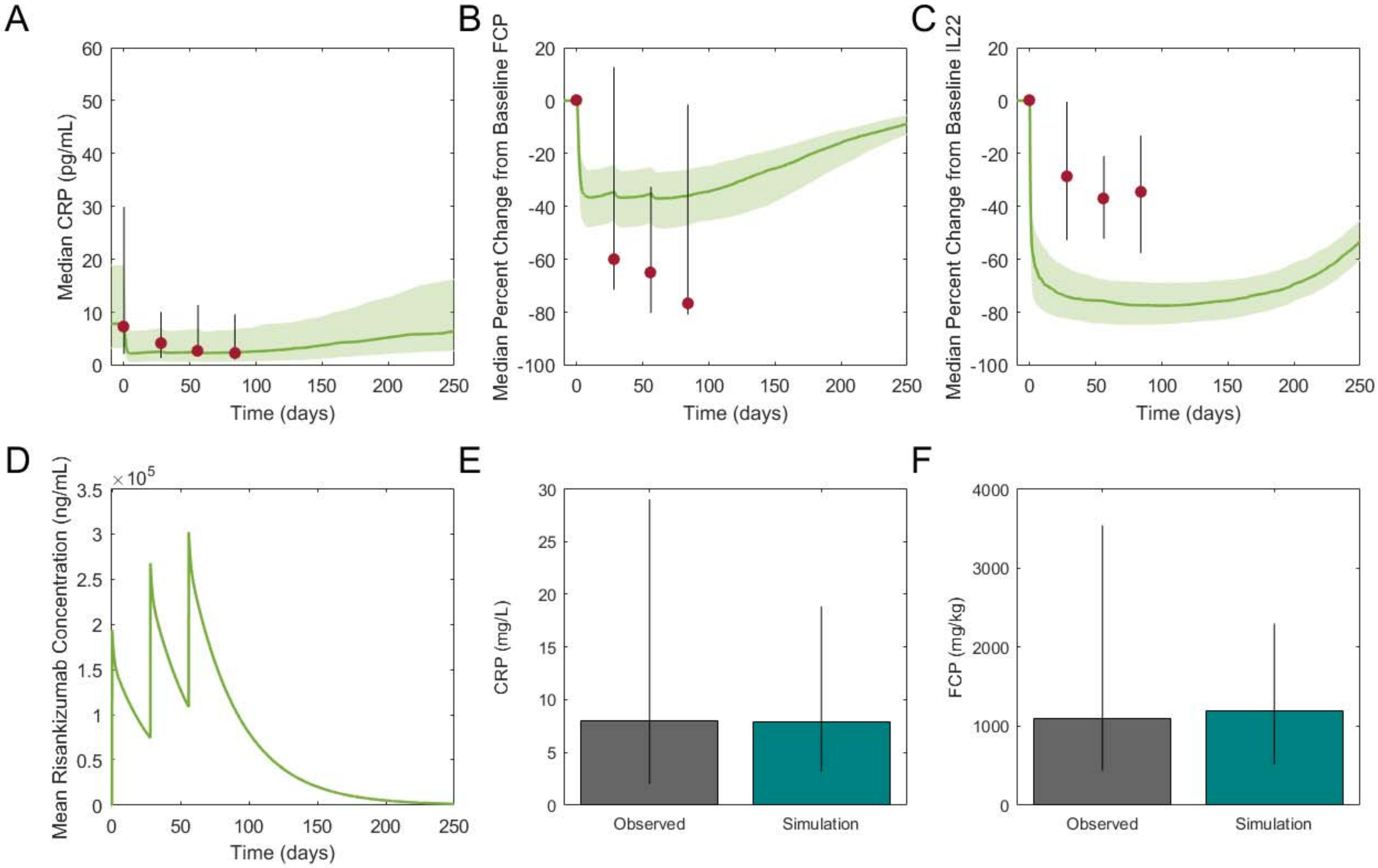

Figure S2: concentration time profiles (CRP, FCP, IL22, risankizumab) in response to a dose of risankizumab (600 mg).

Supplementary Figure 3 (S3)

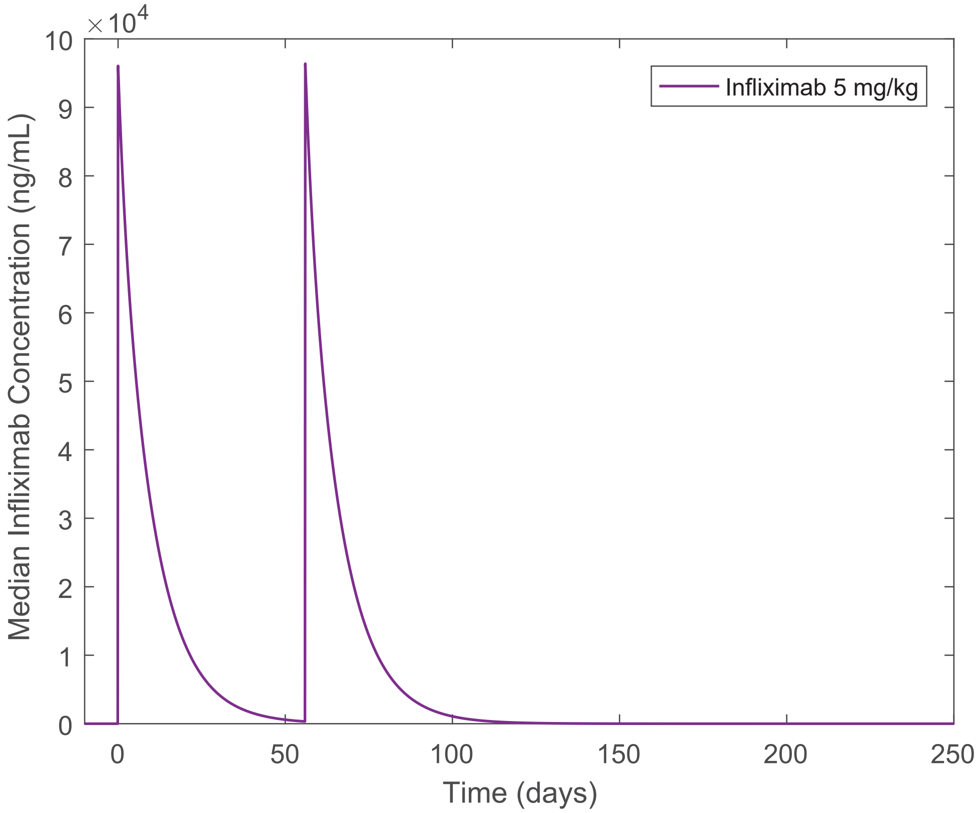

Figure S3: Concentration time profile for 5 mg/kg infliximab given IV at 0 and 8 weeks.

Supplementary Figure 4 (S4)

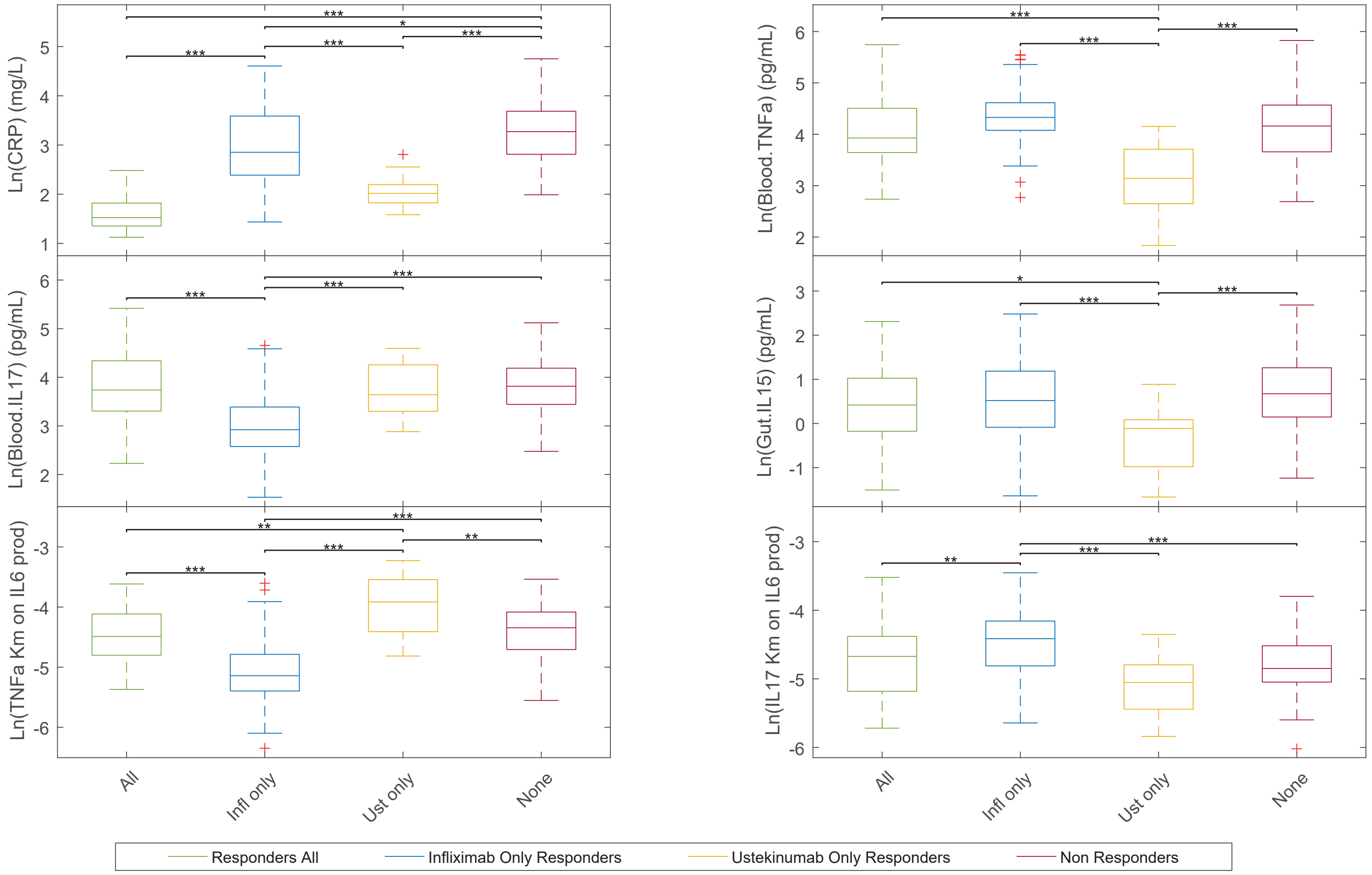

Figure S4: Boxplots of different CRP responder groups when response is considered as an absolute cutoff of 3 mg/L CRP.

Supplementary Figure 5 (S5)

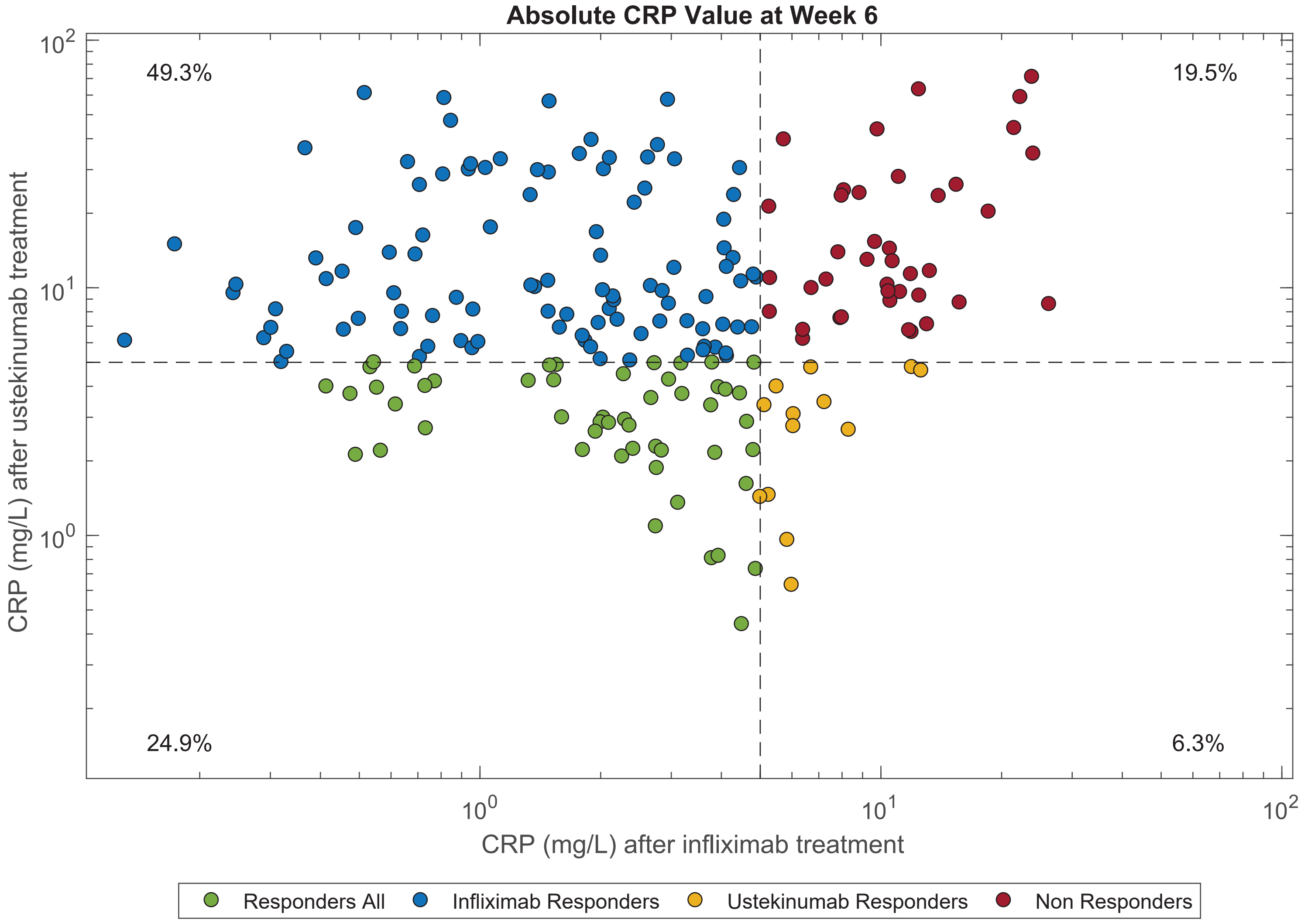

Figure S5: Subject level CRP response to infliximab or ustekinumab using a 5 mg/L CRP cutoff.

Supplementary Figure 6 (S6)

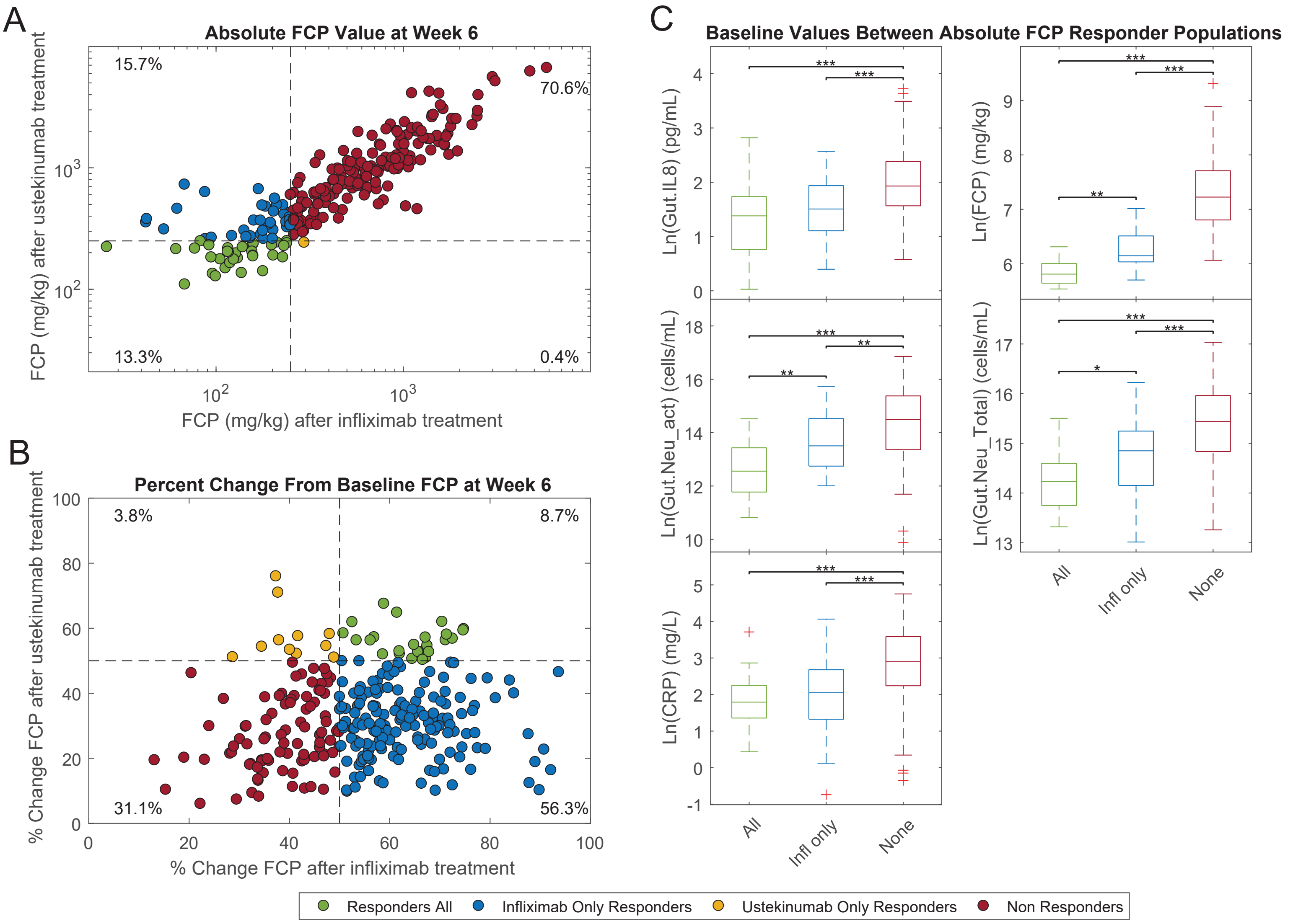

Figure S6: .Subject level FCP response to infliximab or ustekinumab.

Supplementary Figure 7 (S7)

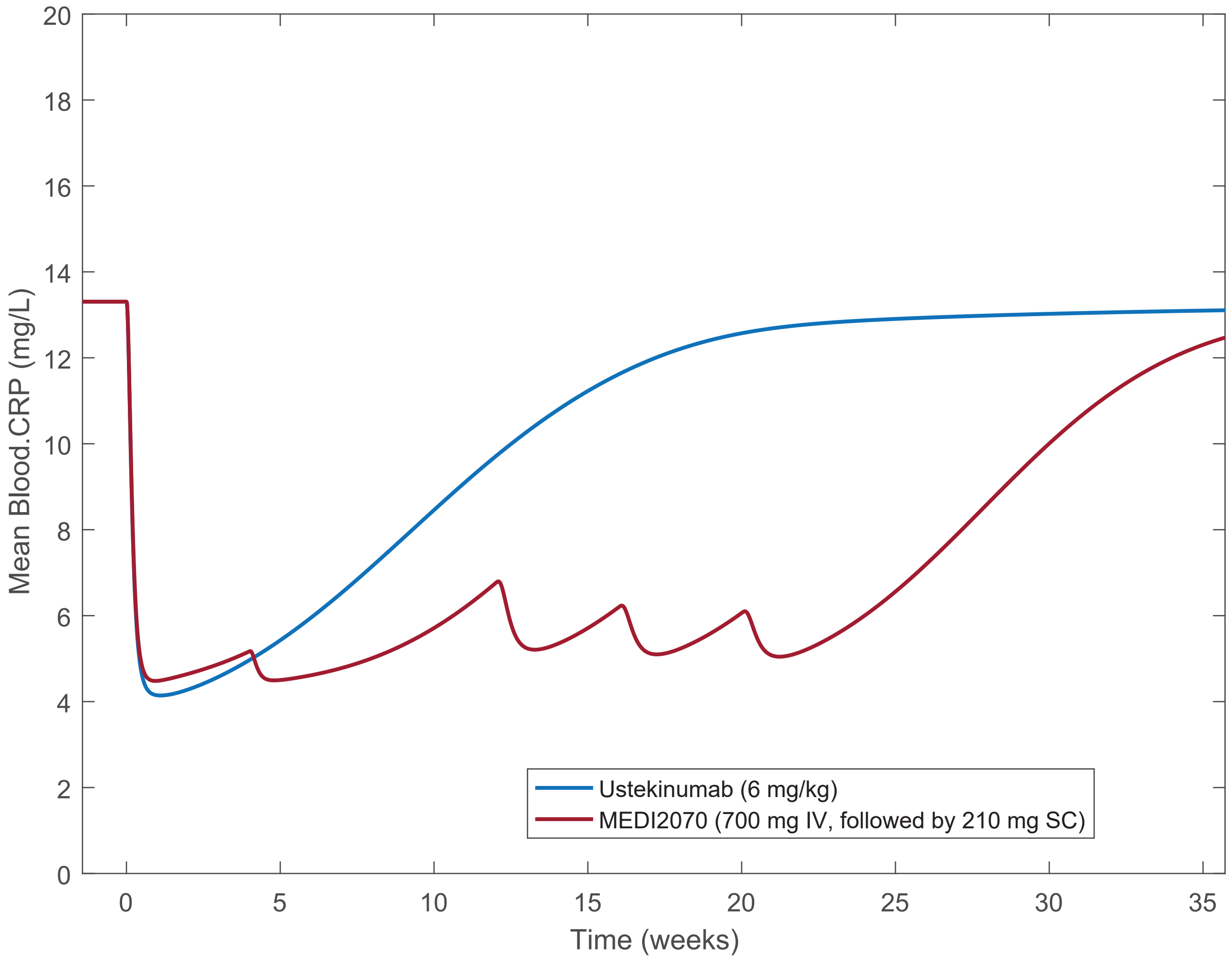

Figure S7: – Prediction of mean CRP profiles following either ustekinumab or MEDI2070 using the same virtual population.

Supplementary Figure 8 (S8)

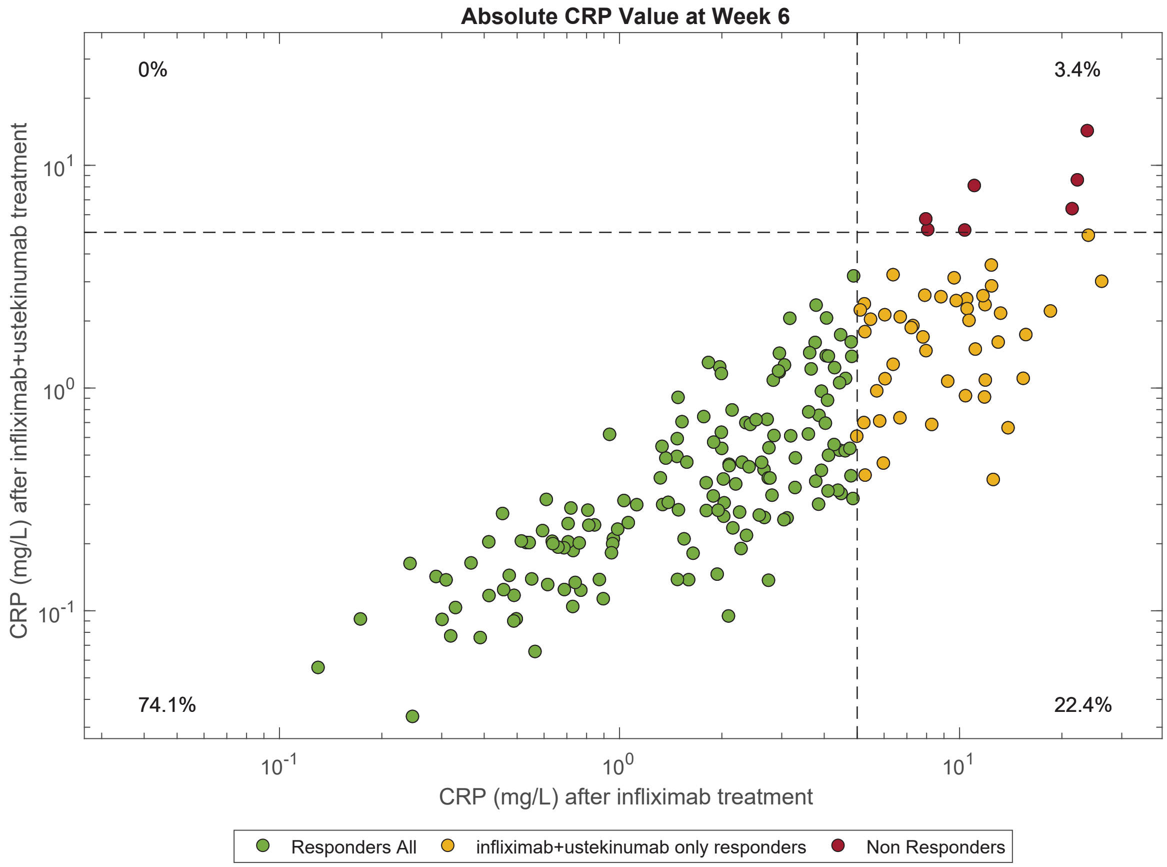

Figure S8: Subject level CRP response to infliximab or ustekinumab + infliximab using a 5 mg/L CRP cutoff.

Supplementary Figure 9 (S9)

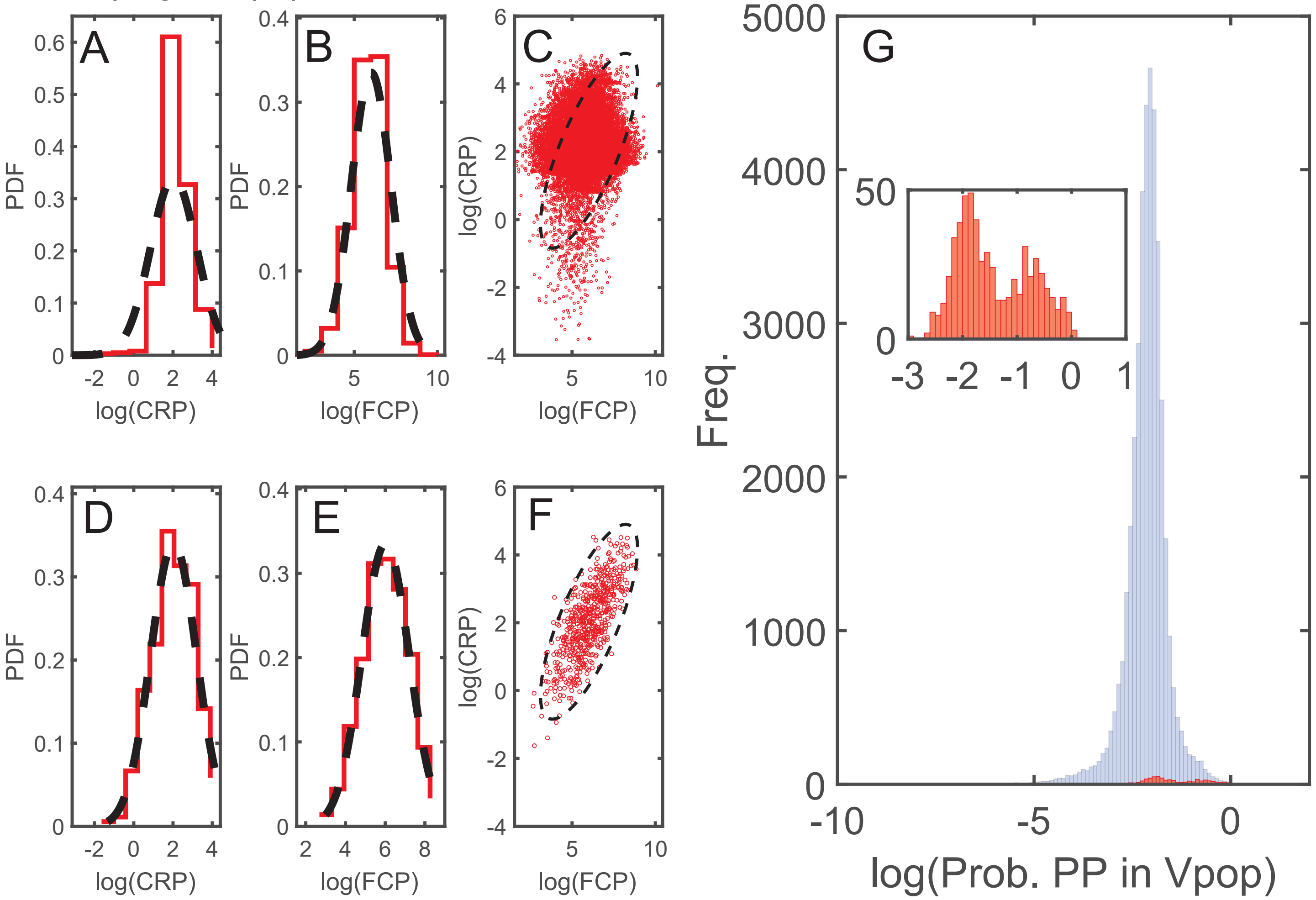

Figure S9: Selection of baseline ustekinumab virtual population.

Supplementary Figure 10 (S10)

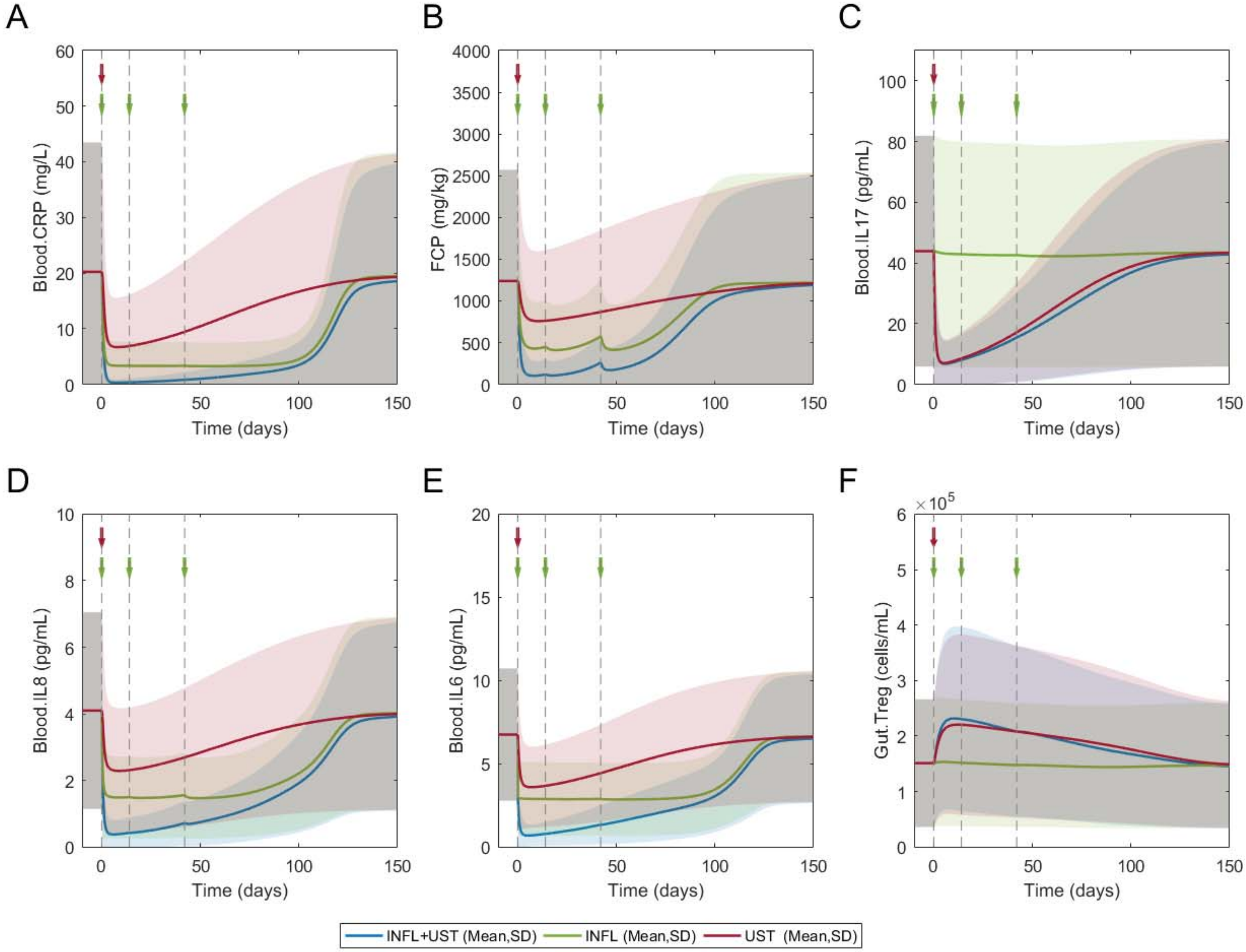

Figure S10: Model prediction of infliximab and ustekinumab combination treatment in CD patients with variability
