# Supplementary material for: A Dynamic Quantitative Systems Pharmacology Model of Inflammatory Bowel Disease: Part 2 – Application to Current Therapies in Crohn’s Disease
Source: Clin Transl Sci. 2020 Aug 21;14(1):249–59. doi: 10.1111/cts.12850 (PMC7877864; doi:10.1111/cts.12850)
Supplement: Supplementary file 2 — Table S1 [file CTS-14-249-s002.docx]

| **Drug** | **Dose** | **Drug Type** | **Trial** | **N** | **N for Model** | **Inclusion Criteria** | **Pubmed ID** |
| --- | --- | --- | --- | --- | --- | --- | --- |
| Ustekinumab | IV: 6 mg/kg at 0 weeks IV: 130 mg at 0 weeks* | Anti-IL12p40 | NCT01369342 | 209, 209 | 1127 | anti-TNF naïve, CDAI 220 - 450 and either CRP > 0.3 mg/L, fecal calprotectin > 250 mg/kg, or endoscopic evidence of inflammation | 27959607 |
| MEDI2070 | IV: 700 mg at 0, 4 weeks then 210 mg SC every 4 weeks from week 12 | Anti-IL23A | NCT01714726 | 59 | 246 | CDAI 220 - 450 and either CRP ≥ 5 mg/L, calprotectin ≥ 250 ug/g, or endoscpoic findings of at least 3 non-anastomotic ulcerations | 28390867 |
| Risankizumab | IV: 200 mg at 0, 4, and 8 weeks IV: 600 mg at 0, 4, and 8 weeks* | Anti-IL23A | NCT02031276 | 41, 41 | 133 | CDAI 220 - 450 and CDEIS of at least 7 | 28411872 |
| Infliximab | IV: 5 mg/kg at 0, 2, and 6 weeks | Anti-TNFα |  | 22 | 144 | CDAI range 153 - 337 | 21461070 |
|  | IV: 5 mg/kg at 0 and 8 weeks | Anti-TNFα |  | 15**** |  | CD patients with acute flare, chronic active disease, or rapid reoccurrence of disease postoperatively | 18484671 |
|  | IV: 5 mg/kg at 0, 2, and 6 weeks*** | Anti-TNFα | ACCENT 1 | 580 |  | CDAI 220-400 | 21741088 |
| PF-04236921 | SC: 10 mg, 50 mg**, or 200 mg at 0, 4 weeks | Anti-IL6 | NCT01287897 | 68, 71, 40 | 286 | CDAI 220 - 450 with CRP ≥ 5 mg/L and ulceration demonstrated by colonoscopy | 29247068 |
|  |  |  |  |  |  |  |  |
| *These cases are shown in supplementary | | |  |  |  |  |  |
| ** Baseline Population shown is chosen from 50 mg group | | | |  |  |  |  |
| *** Concentration is the one show in Baseline Population figure | | | |  |  |  |  |
| **** In the study one patient received adalimumab induction dose 80 mg s.c. at week 0, followed by 40 mg s.c. every other week until week 8 | | | | | | | |
|  |  |  |  |  |  |  |  |
