# Supplementary material for: A Dynamic Quantitative Systems Pharmacology Model of Inflammatory Bowel Disease: Part 2 – Application to Current Therapies in Crohn’s Disease
Source: Clin Transl Sci. 2020 Aug 21;14(1):249–59. doi: 10.1111/cts.12850 (PMC7877864; doi:10.1111/cts.12850)
Supplement: Supplementary file 3 — SupplementaryMaterial S1 [file CTS-14-249-s003.docx]

**Part 2 Supplementary Material**

Main Article – A dynamic quantitative systems pharmacology model of inflammatory bowel disease: Part 2 – application to current therapies in Crohn’s disease

The supplementary material enclosed herewith contains the following:

1. Table S1 – A table of the clinical trials used for the manuscript.
2. Figure S1 shows concentration time profiles (CRP, FCP, ustekinumab) in response to a dose of ustekinumab (130 mg).
3. Figure S2 shows concentration time profiles (CRP, FCP, IL22, risankizumab) in response to a dose of risankizumab (600 mg).
4. Figure S3 - Concentration time profile for 5 mg/kg infliximab given IV at 0 and 8 weeks.
5. Figure S4 – Boxplot of different CRP responder groups when response is considered as an absolute cutoff of 3 mg/L CRP.
6. Figure S5 – Subject level CRP response to infliximab or ustekinumab using a 5 mg/L CRP cutoff.
7. Figure S6 - Subject level FCP response to infliximab or ustekinumab.
8. Figure S7 – Prediction of mean CRP profiles following either ustekinumab or MEDI2070 using the same virtual population.
9. Figure S8 – Subject level CRP response to infliximab or ustekinumab + infliximab using a 5 mg/L CRP cutoff.
10. Figure S9 - Selection of baseline ustekinumab virtual population.
11. Figure S10 - Model prediction of infliximab and ustekinumab combination treatment in CD patients with variability.

**Table 1:** ClinicalTrialsTable.xlsx – contains drug, dose, type, trial number, number of subjects from trial, number of subjects for virtual population, inclusion criteria and PubMed ID

**Supplementary Figure 1:** Biomarker profiles for ustekinumab 130 mg dosing arm. Shown is median change from baseline of (A) CRP and (B) FCP following a single 130 mg IV dose of ustekinumab. (C) Model simulation versus observed ustekinumab concentration time profiles. Median and interquartile range of baseline values for (D) CRP and (E) FCP in the model virtual patient population (green) and observed population (grey) for the UNITI-2 trial. Solid lines indicate median and shaded area indicates interquartile range for the simulation population (N = 1127). Virtual population is the same as in the previous 6 mg/kg ustekinumab case.

**Supplementary Figure 2:** Biomarker profiles for the risankizumab 600 mg dosing arm. (A) Predicted absolute median and interquartile range CRP concentration profile for virtual population compared to observed. Median percent change in (B) FCP and (C) IL22 following risankizumab. (D) Simulated risankizumab concentration time profile of 600 mg dose IV at 0, 4, and 8 weeks. (E,F) Comparison of baseline biomarker values for virtual population (green) versus observed clinical trial population (grey) for the 600 mg dose. Solid lines indicate median and shaded area indicates interquartile range for the simulation population (N = 236).

**Supplementary Figure 3:** Alternative infliximab dosing schedule. Shown is the simulated median infliximab concentration time profile for two 5 mg/kg doses given IV at weeks 0 and 8. Weight assumed as 70 kg.

**Supplementary Figure 4:** Differences between groups using absolute cutoff of 3 mg/L CRP at 6 weeks. Shown are baseline protein levels and parameter values for the 4 groups of percent CRP response. The groups are CRP responders to both drugs (green), responders to infliximab (blue), responders to ustekinumab (orange), and non-responders (red).

**Supplementary Figure 5:** Subject level response to infliximab (5 mg/kg) and ustekinumab (6 mg/kg) at 6 weeks using a 5 mg/L CRP cutoff. Shown is absolute CRP concentration at week 6 following either infliximab (IV at 0, 2, and 6 weeks) or ustekinumab (IV at time 0) administration for each individual virtual patient with values above 5 mg/L at baseline (N = 205). Each dot represents a virtual patient from the virtual population and is the same as in the infliximab only case.

**Supplementary Figure 6:** Subject level FCP response to infliximab (5 mg/kg IV at week 0, 2, and 6) and ustekinumab (single does 6 mg/kg IV) at week 6 in CD patient. Panel A shows the absolute FCP levels at week 6 and Panel B shows the percent change from baseline FCP following either infliximab  or ustekinumab administration to each individual virtual patient (N = 286). In Panel A only patients whose baseline FCP level was above 250 mg/kg (N = 248) were included. Each dot represents a virtual patient and are colored depending upon if they were a FCP responder to both drugs (green), responders to infliximab (blue), responders to ustekinumab (orange), and non-responders (red). Response is defined as > 50% change from baseline in the percent change case and < 250 mg/kg in the absolute value case. Panel C shows bar charts of baseline values for various biomarkers according to the subjects response to infliximab or ustekinumab treatment based on achiever an absolute value of < 250 mg/kg FCP at week 6.Ustekinumab only group was excluded from boxplot because N = 1.

**Supplementary Figure 7:** Prediction of mean CRP profiles following treatment with ustekinumab or MEDI2070. The mean CRP response following single induction dose of ustekinumab (6 mg/kg) or induction and maintenance treatment of MEDI2070 (700 mg/210 mg) for the same virtual population (N = 1127).

**Supplementary Figure 8:** Subject level CRP response to infliximab (5 mg/kg) and ustekinumab (6 mg/kg) plus infliximab combination at 6 weeks. Absolute CRP concentration after 6 weeks of treatment for each individual virtual patient where baseline CRP level is above 5 mg/L (N = 205). Each dot represents a virtual patient from the virtual population and is the same as in the infliximab only case.

**Supplementary Figure 9:** Selection of Baseline Ustekinumab Population. Comparison of the plausible population (N = 40,000) with (a-b) multivariate distribution of CRP and FCP from the UNITI-2 6 mg/kg arm (covariance was assumed similar to all internal data) and (c) 2D projection of the 95% confidence surface of the estimated probability density function. Comparison of the virtual population subset (N = 1127) with (d-e) multivariate distribution of CRP and FCP from the study (covariance was assumed similar to all internal data) and (f) 2D projection of the 95% confidence surface of the estimated probability density function. (g) Histogram of the plausible population (PP) selection probability, where red is the virtual population (Vpop) selected.

**Supplementary Figure 10:** Model prediction of infliximab and ustekinumab combination treatment in CD patients with variability. Mean CRP, FCP, IL17, IL8, IL6, and Treg response to the combination (blue), infliximab only (green), or ustekinumab only (red). Virtual population size is 286 and is the same as in the infliximab only case. Solid lines denote mean response and shaded area denotes standard deviation. Arrows and dotted lines denote dosing of infliximab (green) and ustekinumab (red).

**Drug Interactions Included in the Model:**

**All drugs included bind to the soluble ligand and therefore were modeled using the following differential equations for the complex.**

For anti-IL12p40:

$$\frac{dIL12\_antiIL12p40}{dt}=k_{on_{IL12}}*IL12*antiIL12p40-k_{off_{IL12}}*\left[ IL12\_antiIL12p40 \right]-k_{el_{complex}}\left[ IL12\_antiIL12p40 \right]$$

$$\frac{dIL23\_antiIL12p40}{dt}=k_{on_{IL12}}*IL23*antiIL12p40-k_{off_{IL12}}*\left[ IL23\_antiIL12p40 \right]-k_{el_{complex}}\left[ IL23\_antiIL12p40 \right]$$

For anti-IL23:

$$\frac{dIL23\_antiIL23}{dt}=k_{on_{IL23}}*IL23*antiIL23-k_{off_{IL23}}*[IL23\_antiIL23]-k_{el_{complex}}\left[ IL23\_antiIL23 \right]$$

For anti-TNFa:

$$\frac{dTNFa\_antiTNFA}{dt}=k_{on_{TNFa}}*TNFa*antTNFa-k_{off_{TNFa}}*[TNFa\_antiTNFa]-k_{el_{complex}}\left[ TNFa\_antiTNFa \right]$$

For anti-IL6:

$$\frac{dIL6\_antiIL6}{dt}=k_{on_{IL6}}*IL6*antiIL6-k_{off_{IL6}}*[IL6\_antiIL6] -k_{el_{complex}}\left[ IL6\_antiIL6 \right]$$
